# Population genetics of *Trypanosoma brucei* circulating in *Glossina palpalis palpalis* and domestic animals of the Fontem sleeping sickness focus of Cameroon

**DOI:** 10.1186/1756-3305-7-156

**Published:** 2014-04-01

**Authors:** Gustave Simo, Guy Roger Njitchouang, Tresor Tito Tanekou Melachio, Flobert Njiokou, Gerard Cuny, Asonganyi Tazoacha

**Affiliations:** 1 Department of Biochemistry, Faculty of science, University of Dschang, PO Box 67, Dschang, Cameroon; 2 Department of Animal Biology and Physiology, Parasitology and Ecology Laboratory, Faculty of Science, University of Yaounde I, P.O. Box 812, Yaounde, Cameroon; 3 Institut de Recherche pour le Développement, Unité Mixte de Recherche 177 IRD-CIRAD, Campus International de Baillarguet, TA A17/G, 34398 Montpellier Cedex 5, France; 4 Faculty of medicine and Biomedical Science, University of Yaoundé 1, Yaounde, Cameroon

**Keywords:** *Trypanosoma brucei*, Tsetse flies, Domestic animals, Microsatellite markers, Population genetics

## Abstract

**Background:**

Human African Trypanosomiasis is still a public health threat in Cameroon. To assess *Trypanosoma brucei* strains circulating in the Fontem sleeping sickness focus, we conducted a genetic structure study using microsatellites to assess genotypes circulating in both tsetse flies and domestic animals.

**Method:**

For this study, pyramidal traps were set up and 2695 tsetse flies were collected and 1535 (57%) living flies were dissected and their mid-guts collected. Furthermore, blood samples were collected from 397 domestic animals (pigs, goats, sheep and dogs). DNA was extracted from midguts and blood samples, and specific primers were used to identify trypanosomes of the subgenus *Trypanozoon*. All positive samples were genetically characterized with seven microsatellite markers.

**Results:**

Seventy five (4.7%) midguts of tsetse flies and 140 (35.2%) domestic animals were found infected by trypanosomes of the subgenus *Trypanozoon*. The genetic characterization of 215 *Trypanozoon* positive samples (75 from tsetse and 140 from animals) revealed a genetic diversity between *Trypanosoma brucei* circulating in tsetse and domestic animals. Of these positive samples, 87 (40.5%) single infections were used here to investigate the population genetics of *Trypanosoma brucei* circulating in tsetse and domestic animals. The dendrogram illustrating the genetic similarities between *Trypanosoma brucei* genotypes was subdivided into four clusters. The samples from tsetse belonged to the same cluster whereas the samples from domestic animals and espcially pigs were distributed in the four clusters.

**Conclusion:**

Pigs appeared as the animal species harboring the highest number of different *Trypanosoma brucei* strains. They may play an important role in the propagation of different genotypes. The *F*_ST_ values revealed a sub structuration of *Trypanosoma brucei* according to hosts and sometimes villages. The data obtained from this study may have considerable importance for the understanding of the transmission and the spread of specific genotypes of *Trypanosoma brucei*.

## Background

Human African Trypanosomiasis (HAT) also known as sleeping sickness is an important public health disease in sub-Saharan Africa. About 60 million people are at risk, with 10 000 new cases reported yearly, with new estimated cumulative infection cases of about 50 000 to 70 000 [1]. The causative agent of HAT is a protozoan parasite belonging to *Trypanosoma brucei* (*T. brucei*) species. This parasite is transmitted by tsetse flies of the genus *Glossina. T. brucei* infects human as well as a variety of domestic and wild animals in sub-Saharan Africa. Three subspecies of *T. brucei* are currently recognized [2]: *Trypanosoma brucei brucei* (*T. b. brucei*), which is defined as infecting animals but not humans and is present throughout the tsetse distribution area in Africa; *Trypanosoma brucei gambiense* (*T. b. gambiense*), which is infective for humans in West and Central Africa and whose infection results in chronic human sleeping sickness; and *Trypanosoma brucei rhodesiense* (*T. b. rhodesiense*), which is defined as human infective and localized in East and South Africa and causes the acute form of sleeping sickness.

During the last few decades, there has been considerable effort to genetically characterize *T. brucei* isolated from vertebrates and tsetse flies in order to understand the impact of the genetic diversity of trypanosomes on the epidemiology of HAT. Most of these studies were performed on parasite strains isolated from tsetse and mammals. From such strains, unique isolates were obtained and their complete genotypes were easily obtained. However, during the isolation process, a sub sampling of existing genetic diversity occurs inside the host [3], resulting in a possible information loss. The need to undertake investigations on field samples without isolation was a challenge until recently when microsatellite markers were developed. The microsatellite DNA sequences are simple sequence repeats (SSRs), which are hyper-variable, ubiquitous and co-dominant [4]. They occur randomly and abundantly in eukaryotic genomes [5] and are widely used in genetics and phylogenetic studies [4,6]. For the subgenus *Trypanozoon*, a panel of several microsatellite markers has been identified during the last two decades. These markers have been widely used for the molecular characterization of trypanosomes, the assessment of the population structure and reproductive mode [3,7-9], and the construction of the genetic map of trypanosomes [10,11]. The higher sensitivity and specificity of these markers enabled us to genetically characterize *T. brucei* directly from biological samples such as tsetse mid-guts, blood, cerebrospinal fluid and lymph [3,12-14]. Using these biological fluids, the microsatellite markers provided data that permitted us to better understand some biological, genetic and epidemiological aspects of HAT. For instance, the microsatellite markers revealed higher levels of mixed infections of different *T. brucei* genotypes in tsetse mid-guts [12]. They also revealed a genetic diversity between strains of *T. b. gambiense* circulating in different fluids of the same HAT patient [13] and multiple infections of *T. b. gambiense* in the blood and cerebrospinal fluid of HAT patients of Angola [14]. Despite the considerable number of data generated from the molecular characterization of *T. brucei,* few investigations have been undertaken to characterize the trypanosomes circulating in tsetse flies and mammals of the same locality. It is obvious that such investigations are important to better understand the circulation, the transmission and the spread of different genotypes of trypanosomes.

During the last decade, investigations on the genetic characterization of *T. brucei* circulating in tsetse flies and domestic animals of the Fontem sleeping sickness focus of Cameroon revealed a wide range of *T. brucei* genotypes as well as a high number of mixed genotypes of *T. brucei* in tsetse flies and domestic animals [12,15]. Despite the genetic diversity revealed by these studies, our knowledge of the transmission of different genotypes between trypanosome hosts remains incomplete. Detailed knowledge on the trypanosome genotypes that circulate between tsetse and mammals appears crucial for a better understanding of the population genetics, transmission, and distribution of *T. brucei* genotypes in different hosts and also for control operations.

For this study, single infections of *T. brucei* were extracted from data published by Simo *et al.*[12,15] and then analyzed in order to improve our knowledge on the population genetics of this parasite, and to understand the transmission as well as the circulation of different *T. brucei* genotypes between tsetse flies and domestic animals of the Fontem sleeping sickness focus of Cameroon.

## Methods

### Study area

The Fontem sleeping sickness focus (05°40′12″N, 09°55′33″E) is located in the Lebialem, Manyu and Koupe-Manengouba Divisions of the southwest region of Cameroon. This focus is characterized by a tropical humid climate with varied topography of hills and valleys through which several high speed rivers flow. This focus is subdivided into three sub foci: the north, the center and the south sub foci [16]. The main population activities are subsistence agriculture, palm oil extraction, animal husbandry and small scale poultry farming. The dense population of humans, domestic animals and tsetse flies are found scattered in the pre-forest/forest vegetation of the valleys and hills [17]. *Glossina palpalis palpalis* is the main vector of HAT in this focus [18]. Several domestic animal species including dog, pigs, sheep, and goat are found in this focus.

### Entomological surveys

For this study, two entomological surveys were carried out in 5 villages (Besali, Bechati, Folepi, Agong and Menji) of the Fontem HAT focus [12]. The first survey was performed in November 2006 and the second in April 2007. In each village, pyramidal traps [19] were deployed in various tsetse fly favorable biotopes. The trapping procedure and the dissection of flies were performed as described in Simo *et al.*[12].

### Blood collection and parasitological analyses

The domestic animals were sampled during two field surveys in the Fontem HAT focus: the first survey was performed in July 2006 and the second in June 2007. The sampling was carried out in eight villages: Besali, Bechati, Folepi and Agong in the northern sub focus and Nsoko, Fossung, Menji and Azi in the central sub focus [15]. In each village, all domestic animals that had spent at least 3 months in the study zone were selected. All pigs and dogs sampled in this study were of local breed, originating from a mixture of different breeds. The sheep and goats were dwarf breeds. Some pigs were kept in pigsties whereas the others, as well as the other animals were allowed to move freely around the villages.

Sensitization, the blood collection procedures and the parasitological tests have been already described in Simo *et al.*[15].

### DNA extraction

In the laboratory, the alcohol used to preserve tsetse mid-guts contained in micro-tubes was evaporated for 60 minutes in an oven set at 80°C. Subsequently, 300 μl of Chelex 5% [20] was added to each tube and the mixture vortexed for 10 minutes. Thereafter, the tubes were incubated first at 56°C for one hour, and then at 100°C for 30 minutes. The tubes were centrifuged at 14 000 rpm for 10 minutes and the supernatant (DNA extract) was collected and stored at −20°C until used.

For the blood samples collected in domestic animals, DNA was extracted from 1 ml of blood using the kit “DNeasy Tissue kit” (Qiagen) as described by Simo *et al.*[15]. The DNA extract was used directly for PCR or stored at −20°C.

### Identification of trypanosomes belonging to *Trypanozoon*

This identification was performed using the *Trypanozoon* specific primers TBR1/2 [21], as described by Simo *et al.*[16]. The amplification reactions were performed in a final volume of 25 μl containing 5 μl of DNA extracts. At the end of each amplification reaction, 10 μl of each amplified product was resolved on 2% agarose gel. All TBR1/2 positive DNA samples were used further for the subsequent genetic characterization by microsatellite markers.

### Genetic characterization of TBR1/2 positive DNA samples using microsatellite DNA markers

All TBR1/2 positive DNA samples (originating from tsetse flies and domestic animals) were genetically characterized using seven microsatellite markers [3,7]. For the samples originating from domestic animals, the amplification reactions were performed in a final volume of 15 μl containing 3 μl of DNA extract whereas for samples coming from tsetse mid-guts, the amplification reactions were performed in a final volume of 25 μl containing 5 μl of DNA extract. Whatever the origin of DNA samples, the amplifications were performed in two rounds for the M6C8 and MT3033 markers; the first round was done as described by Biteau *et al.*[7]. During the second round, 1 μl of the amplified product of the first PCR was used as DNA template. For the other markers (Micbg1, Micbg5, Micbg6, Misatg4 and Misatg9), the amplification conditions were identical to those described by Koffi *et al.*[3]. The amplified products were checked by electrophoresis on 2% agarose gel and thereafter, the allelic profiles were obtained through an electrophoresis on 10% non-denaturing acrylamide gels as described in Simo *et al.*[12,15].

A sample showing more than two alleles was considered here as having multiple infections because *T. brucei* species is a diploid organism and must have one allele or one DNA fragment (homozygote) or two alleles (heterozygote) after the resolution of the amplified products of each microsatellite locus.

### Data analysis

For this analysis, the samples showing more than two alleles or having multiple genotypes were excluded. We considered here as samples with multiple genotypes all samples for which at least three alleles (three DNA fragments) were identified for at least one microsatellite marker. The genetics structure within *T. brucei* populations circulating in tsetse flies and domestic animals was assessed through Wright’s F-statistics [22]. *F*_ST_ is a measure of deviation from random distribution of individuals between subsamples (and thus, populations’ differentiation). For the differentiation analysis of the populations, the total sample (87 individuals) was first subdivided into five subsamples; each subsample corresponding to one of the five hosts of trypanosomes (tsetse, dog, sheep, goat and pig). Thereafter, a second subdivision into eight subsamples was performed; each subsample here corresponding to villages where tsetse flies and animals were sampled. Wright’s F-statistics were estimated using Weir and Cockerham’s unbiased estimators [23] in Fstat 2.9.4 software ([24] updated from Goudet [25]). *F*_ST_ is a convenient measure of differentiation among the different subsamples of a data set. Its estimator is expected around 0 under the null hypothesis of random distribution of genotypes across sub-samples. On the contrary, *F*_ST_ estimator displays positive values, up to 1, in case of genetic differences. For *F*_ST,_ the estimator was *Ө* and its significance tested through 10000 permutations of individuals between subsamples. To get an overall idea of the distribution of individuals across hosts and villages, an unrooted NJTREE was computed by the MEGA 3.1 software [26], using the Cavalli-Sforza and Edwards [27] chord distances matrix, which were computed in the GENETIX version 4.05 software package.

The genetic diversity within *T. brucei* populations that circulate in tsetse flies and domestic animals was measured with Nei’s unbiased estimator Hs for each locus, over all loci and for multilocus genotypes (MLGs) [28]. All tests and population genetic measures were undertaken with Fstat 2.9.4 ([24], updated from Goudet [25]). All differentiation tests were repeated with the multilocus genotypes (MLGs) where each MLG is coded as an allele of a unique locus as described by Nébavi *et al.*[29].

### Ethical approval

This study was carried out in strict accordance with the recommendations in the Guide for the Care and Use of Animals of the Department of animal biology and physiology of the Faculty of Science of the University of Yaoundé 1 of Cameroon.

## Results

This study involves 2695 tsetse flies caught in five villages and 397 domestic animals sampled in eight villages.

### Identification of *Trypanozoon* in tsetse mid-guts and their genetic characterization

During two entomological surveys, 2695 *Glossina palpalis palpalis* were caught and a total of 1596 (59.2%) flies were dissected and 352 teneral flies identified. Details concerning entomological results can be found in Simo *et al.*[12]. The specific PCR targeting a multi-copy 177 bp repeat sequence of trypanosomes of the subgenus *Trypanozoon* revealed 75 (4.7%) mid-guts infected by trypanosomes of this subgenus. The genetic characterization was performed on these 75 *Trypanozoon* positive samples with seven microsatellite markers. Only 5 (Micbg1, M6C8, MT3033, Misatg4 and Misatg9) of the 7 microsatellite markers showed amplifications; no amplification for all the 75 *Trypanozoon* positive samples was obtained for the markers Micbg5 and Micbg6 [12]. The characteristics of each sample and the allele size for each marker are reported in Simo *et al.*[12]. Of the 75 samples analyzed, about 41.3% were multiple infections and therefore, were not considered in the present study since they cannot be included in the population genetic studies. The current study targets 44 samples (single infections) for which no more than two alleles were observed (Table 1). Details concerning the characteristics of each of the 44 samples and the allelic profiles for each marker are reported in Table 1.

**Table 1:** **Characteristics of mid-gut single infections of *T. brucei *** **and size of alleles at each microsatellite locus**

**Sample code**	**Villages**	**Tsetse species**	**Size of alleles at each microsatellite locus**					
			**Sex**	**M6C8**	**MT3033**	**Micbg1**	**Misatg4**	**MisatG9**
T2261	Bechati	G. pal	F	115/105	162/142	150/166	136/128	000/000
T2259	Bechati	G. pal	F	115/105	162/142	150/136	136/128	000/000
T2310	Bechati	G. pal	M	115/105	162/142	150/136	136/128	170/170
T2262	Bechati	G. pal	M	115/105	162/142	150/136	136/128	170/170
T105	Bechati	G. pal	F	115/105	162/142	150/136	136/128	172/170
T177	Bechati	G. pal	M	115/105	162/142	150/136	136/128	172/170
T2180	Bechati	G. pal	F	115/105	162/142	150/136	136/128	170/086
T2362	Bechati	G. pal	F	115/105	162/142	150/136	136/128	170/170
T2271	Bechati	G. pal	F	115/105	162/142	150/136	136/128	170/170
T2247	Bechati	G. pal	M	115/105	162/142	150/136	136/128	170/170
T2205	Bechati	G. pal	F	115/105	162/142	150/136	136/128	170/170
T35	Besali	G. pal	M	091/085	166/156	174/162	126/116	172/170
T39	Besali	G. pal	F	091/085	166/156	174/162	126/116	172/170
T239	Folepi	G. pal	M	115/105	162/142	150/136	136/128	170/086
T218	Folepi	G. pal	M	115/105	162/142	150/136	136/128	000/000
T1823	Folepi	G. pal	M	115/105	162/142	150/136	136/128	000/000
T1075	Folepi	G. pal	M	115/105	162/142	150/136	136/128	000/000
T1970	Folepi	G. pal	F	115/105	162/142	150/136	136/128	170/170
T306	Folepi	G. pal	F	115/105	162/142	150/136	136/128	170/170
T316	Folepi	G. pal	F	095/083	162/142	150/136	136/128	172/170
T345	Folepi	G. pal	M	115/105	162/142	150/136	136/128	170/170
T441	Folepi	G. pal	F	115/105	162/142	150/136	136/128	172/170
T443	Folepi	G. pal	M	115/105	162/142	150/136	136/128	170/170
T357	Folepi	G. pal	M	091/085	166/156	174/162	126/116	000/000
T1855	Folepi	G. pal	F	115/105	162/142	150/136	136/128	000/000
T1344	Folepi	G. pal	M	115/105	162/142	150/136	136/128	000/000
T1005	Folepi	G. pal	F	115/105	162/142	150/136	136/128	000/000
T1539	Folepi	G. pal	F	115/105	162/142	150/136	136/128	172/170
T1710	Folepi	G. pal	M	115/105	162/142	150/136	136/128	170/170
T1688	Folepi	G. pal	F	115/105	162/142	150/136	136/128	000/000
T1947	Folepi	G. pal	F	115/105	162/142	150/136	136/128	000/000
T2066	Folepi	G. pal	F	115/105	162/142	150/136	136/128	000/000
T1954	Folepi	G. pal	F	115/105	162/142	150/136	136/128	170/086
T1506	Folepi	G. pal	F	115/105	162/142	150/136	136/128	172/170
T1743	Folepi	G. pal	M	115/105	162/142	150/136	136/128	000/000
T1347	Folepi	G. pal	M	115/105	162/142	150/136	136/128	172/170
T1653	Folepi	G. pal	F	115/105	162/142	150/136	136/128	172/170
T1646	Folepi	G. pal	F	091/085	162/142	150/136	136/128	136/134
T2029	Folepi	G. pal	F	115/105	162/142	150/136	136/128	172/170
T2093	Folepi	G. pal	F	115/105	162/142	150/136	136/128	170/170
T2445	Menji	G. pal	F	091/085	166/156	174/162	126/116	170/086
T2482	Menji	G. pal	F	115/105	162/142	150/136	136/128	000/000
T2481	Menji	G. pal	F	115/105	162/142	150/136	136/128	170/170
T2427	Menji	G. pal	F	115/105	162/142	150/136	136/128	170/170

*G. pal Glossina palpalis palpalis*; *F* female; *M* male. Results of microsatellite markers are given as: AAA/BBB where AAA is the size in base pair of the largest allele and BBB, the size of the smallest allele. 000/000: no amplification.

### Identification of *Trypanozoon* in domestic animals and their genetic characterization

The 397 domestic animals sampled in this study include 225 pigs, 87 goats, 65 sheep and 20 dogs. The origins of these animals as well as their parasitological status are reported in Simo *et al.*[15]. The specific PCR targeting a multi-copy 177 bp repeat sequence of trypanosomes of the subgenus *Trypanozoon* revealed 140 (35.3%) animals infected by these trypanosomes. Although the primers used here are able to identify all trypanosomes of the subgenus *Trypanozoon*, only *T. b. gambiense* and mainly *T. b. brucei* have been identified in animals and tsetse flies of this region [16,18].

The seven microsatellite markers used for the mid-gut infections were also used for the genetic characterization of the 140 *Trypanozoon* positive samples from animals. Most of the 140 samples were amplified by the seven markers. However, to evaluate the circulation of *T. brucei* genotypes between tsetse and domestic animals, only the 5 markers for which amplified products were obtained for the 75 *Trypanozoon* positive samples originating from tsetse flies were considered here. Of the 140 *Trypanozoon* positive samples originating from domestic animals, 97 were considered as having multiple trypanosome genotypes. Therefore, only 43 samples were considered for this study. The details concerning the characteristics of each of these samples and the size of alleles at each microsatellite locus are reported in Table 2.

**Table 2:** **Characteristics of domestic animals’ single infections of *T. brucei *** **and size of alleles at each microsatellite locus**

**Sample code**	**Animal species**	**Village**	**Microsatellite markers**				
			**M6C8**	**MT3033**	**MICBG1**	**MISATG4**	**MISATG9**
P302	Pig	Agong	175/175	162/142	190/190	190/159	234/086
P303	Pig	Agong	000/000	310/310	190/190	221/221	086/086
P304	Pig	Agong	175/175	162/142	190/190	190/116	234/086
P306	Pig	Agong	175/175	310/310	190/190	221/149	086/086
P309	Pig	Agong	175/175	162/142	190/136	221/149	086/086
P310	Pig	Agong	000/000	162/142	190/190	221/149	234/086
S595	Sheep	Agong	115/150	162/142	190/190	123/116	086/086
S596	Sheep	Agong	115/150	162/142	190/190	221/149	242/242
S602	Sheep	Agong	091/085	208/208	136/136	000/000	242/242
P236	Pig	Bechati	115/105	162/142	200/190	190/136	000/000
P240	Pig	Bechati	175/175	162/142	190/190	190/128	234/234
P245	Pig	Bechati	175/175	310/310	190/190	159/136	086/086
G246	Goat	Bechati	091/085	000/000	150/150	221/221	234/086
P247	Pig	Bechati	175/175	310/310	200/190	128/128	086/086
S255	Sheep	Bechati	275/275	310/200	162/108	116/116	234/234
G281	Goat	Bechati	091/085	162/142	190/108	165/128	086/086
G546	Goat	Bechati	091/085	000/000	190/108	165/128	242/242
G548	Goat	Bechati	091/085	310/200	224/108	165/128	242/242
G550	Goat	Bechati	091/085	162/142	190/108	000/000	086/086
G590	Goat	Bechati	301/301	000/000	162/115	136/128	136/086
G593	Goat	Bechati	091/085	310/200	174/150	000/000	242/242
S219	Sheep	Besali	275/275	310/200	250/190	221/110	234/234
S221	Sheep	Besali	275/275	200/200	234/190	165/165	234/234
P229	Pig	Besali	275/175	200/200	220/190	128/116	234/234
P301	Pig	Folepi	175/175	162/142	190/190	126/126	234/086
D572	Dog	Folepi	000/000	000/000	000/000	000/000	242/176
S587	Sheep	Folepi	115/070	156/142	190/190	000/000	242/242
P676	Pig	Fossung	179/175	310/142	126/126	000/000	086/086
P334	Pig	Menji	175/175	166/156	174/162	221/116	086/086
P335	Pig	Menji	175/175	166/156	200/190	116/116	234/086
P605	Pig	Menji	179/175	310/142	190/190	221/221	242/242
P606	Pig	Menji	091/085	310/142	136/136	149/136	242/234
P624	Pig	Menji	091/085	310/142	136/115	123/128	086/086
P631	Pig	Menji	091/085	310/142	174/162	149/136	086/086
P641	Pig	Menji	091/085	310/142	250/190	221/221	128/086
P643	Pig	Menji	091/085	310/142	174/162	221/221	086/086
P645	Pig	Menji	091/085	310/142	174/162	123/128	128/086
P648	Pig	Menji	179/175	310/142	220/115	221/221	128/086
G353	Goat	Nsoko	275/275	187/156	190/108	221/149	234/086
G355	Goat	Nsoko	275/275	166/156	190/108	221/149	234/086
P658	Pig	Nsoko	179/175	310/142	174/136	149/116	128/086
G665	Goat	Nsoko	091/085	310/142	190/115	000/000	146/146
P672	Pig	Nsoko	179/175	162/142	136/115	123/116	086/086
S390	Sheep	Azi	091/085	200/187	190/108	116/116	234/086

Results of microsatellite markers are given as: AAA/BBB where AAA is the size in base pair of the largest allele and BBB, the size of the smallest allele. 000/000: no amplification.

A total of 215 *Trypanozoon* positive samples were identified in tsetse flies and domestic animals. Of these samples, 87 were single infections. For the five markers considered here, about 71 alleles were identified in both multiple and single infections. Twenty (28%) of these alleles could not be considered for this analysis because they were always found in multiple infections. Most of these alleles were found in a very few number of samples and could thus be considered as belonging to minor genotypes. For instance, alleles such as 158, 145 and 124 of MT3033 were identified only in one sample of multiple infections. It is also the case for allele 75 of M6C8, 90 and 87 of Micbg1.

The number of alleles revealed for each locus was higher in domestic animals than tsetse flies (Table 3). Most alleles found in tsetse were identified in animals. Twelve alleles were identified in tsetse flies and all species of domestic animals. For instance, the alleles such as 142, 156 and 162 of MT3033, 86 of Misatg9, 85 and 91 of M6C8 were identified in tsetse flies and all domestic animal species (Additional file 1: Figure S1). However, some alleles such as 83 of M6C8, 166 of Micbg1, 170 and 172 of Misatg9 were identified only in tsetse flies. Furthermore, some alleles found in domestic animals were not identified in tsetse flies (Additional file 1: Figure S1).

**Table 3:** Number of alleles for each locus and according to different hosts of trypanosomes

	**Number of alleles sampled per population**				
**Loci**	**Tsetse**	**Goat**	**Pig**	**Sheep**	**Total**
MICBG1	5	7	9	6	14
MISATG	4	5	9	6	11
MISATG	5	5	4	3	9
M6C8	6	4	7	6	12
MT3033	4	7	6	7	8
Total	24	28	35	28	54

Eighty-seven single infections of *T. brucei* including 44 originating from tsetse flies and 43 from domestic animals were selected for the genetic analyses (Tables 1 and 2). Genotypes of 87 *T. brucei* positive samples were scored at 5 microsatellite loci. These markers showed some degree of genetic diversity within *T. brucei* populations circulating in tsetse flies and domestic animals of the Fontem HAT focus. The value of the genetic diversity ranges from 0.369 (MISATG locus) to 0.888 (MT3033 locus), with an overall value of 0.695 (Table 4). All the microsatellite markers were highly polymorphic with a substantial number of different multilocus genotypes (MLGs).

**Table 4:** Value of the genetic diversity at each microsatellite locus

**Loci**	**Observed genetic diversity**	**Expected genetic diversity**
MICBG1	0.740	0.710
MISATG	0.756	0.772
MISATG	0.369	0.613
M6C8	0.723	0.708
MT3033	0.888	0.762
Overall	0.695	0.713

### Neighbour-joining analysis

**Figure 1:**
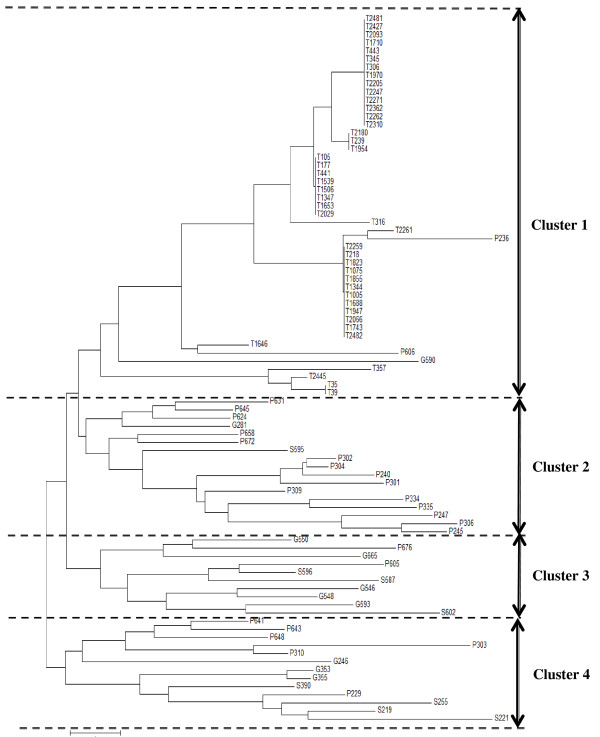
**Dendrogram showing the genetic diversity in *T. brucei *circulating in tsetse flies and domestic animals of the sleeping sickness focus of Fontem.** P: pig; S: sheep; G: Goat; T: Tsetse flies.

To examine the level of the genetic diversity in the population of *T. brucei*, multilocus genotypes were derived for each sample from the genotyping performed with the five microsatellite markers considered for the present study. Thereafter, a dendrogram of similarity was constructed from the data obtained. A total of 40 distinct multilocus genotypes were identified from the 87 samples considered here. The dendrogram generated indicates that the *T. brucei* populations form a large group with a substantial number of different genotypes (Figure 1). This dendrogram can be subdivided into four clusters (Figure 1): the first cluster contains all samples of *T. brucei* originating from tsetse flies and three additional samples coming from pig (P236 and P606) and goat (G590). The second cluster contains 15 samples of *T. brucei* originating from pig and two additional samples coming from goat (G281) and sheep (S595). The third cluster contains samples originating mostly from sheep and goats and the last cluster contains samples coming from different species of domestic animals. Considering the distribution of *T. brucei* genotypes in different clusters, it appears that the trypanosomes’ hosts can be considered as separate entities for investigations aiming to better understand the genetic diversity within *T. brucei* populations. In this light, subsequent analyses were undertaken with subpopulations defined as all *T. brucei* positive samples originating from the same host or coming from the same village.

### Differentiation of *T. brucei* populations

Considering the samples originating from the same host as one subpopulation, the pairwise *F*_ST_ values between different subpopulations (Table 5) indicate a genetic differentiation between the trypanosomes’ hosts (values ranging from 0.0317 to 0.3163). Except for the comparisons between trypanosomes extracted from goat and pig, as well as, goat and sheep, which show a lower genetic differentiation (*F*_ST_ = 0.0797 and 0.0317, respectively), the other comparisons show greater genetic differentiation (Table 5). The genetic differentiation between goat and sheep was not significant (P = 0.1318) whereas the values obtained for the other comparisons were highly significant (P < 0.001). Moreover, the pairwise *F*_ST_ values indicate some genetic differentiations between villages (values ranging from 0.0124 to 0.3080). Some villages show very little genetic differentiation while others reveal greater genetic differentiation with significant p values (Table 6). The results of this study show that *T. brucei* populations of the Fontem HAT focus display diverse genotypes. However, few closely related genotypes circulate most often in tsetse flies.

**Table 5:** FST values between the hosts of trypanosomes

	**Goat**	**Pig**	**Sheep**
G. p. pal	0.2752*	0.3040*	0.3163*
Goat		0.0797*	0.0317
Pig			0.1077

*G.p. pal Glossina palpalis palpalis*; *: P value significant.

**Table 6:** Values of FST between villages

	**Bechati**	**Besali**	**Folepi**	**Menji**	**Nsoko**
Agong	0.1485*	0.2143*	0.3140*	0.0858*	0.0555
Bechati		0.1530*	0.0403	0.0437	0.1061*
Besali			0.3080*	0.1192	0.0700
Folepi				0.1678*	0.2742*
Menji					0.0124

*: *P value* significant.

## Discussion

This study on the population genetics of *T. brucei* revealed a high genetic diversity between *T. brucei* circulating in tsetse flies and domestic animals of the Fontem HAT focus of Cameroon. For this study, multiple infections were excluded because they could not show the genetic information for each individual trypanosome. Such information is needed for population genetic studies like for instance the evaluation of the F statistics. Therefore, some alleles involved in these infections (probably minor genotypes) were not taken into account. Whatever the microsatellite marker, the majority of alleles identified in tsetse flies and domestic animals were selected for this study and consequently, the genotypes analyzed here are probably the major genotypes found most often in the Fontem focus. However, it is important to point out that the exclusion of some alleles and the fact that wild animals (host of trypanosomes and source of blood meals for tsetse flies) were not included in this study have probably led to an underestimation of the real genetic diversity. The identification of *T. brucei* in tsetse flies and domestic animals of the Fontem HAT confirms results of previous authors [16,18] who identified *T. b. brucei* and *T. b. gambiense* in tsetse flies and mammals (human and animals) of this region. The previous identification of *T. b. gambiense* at a very low prevalence in patients (0.05% in human for instance [16]) and domestic animals suggests that most strains analyzed in this study belong to *T. b. brucei*.

For the five microsatellite markers considered here, more alleles were identified in animals compared to tsetse flies (Table 3); thus indicating a high level of genetic diversity in *T. brucei* strains circulating in animals compared to those found in tsetse flies. These results are in line with the observations of MacLeod *et al.*[30] that the full level of *T. brucei* diversity is only apparent when tsetse flies are examined. The fact that some genotypes circulating in animals (minor genotypes) could not be found in tsetse mid-guts is difficult to explain because the mid-gut genotypes (immature infections) probably come from infected animals. Nevertheless, some minor genotypes that cannot be observed in the midgut can develop to maturation in the salivary glands, and therefore, can be transmitted to animals. In such context, the identification of some genotypes in animals, but not in tsetse flies could be explained by the fact that several infected tsetse flies with different *T. brucei* genotypes can feed simultaneously or successively on the same animal [15]. During their blood meals, infected flies can transmit different *T. brucei* genotypes to the vertebrate host. Another explanation may result from the bottlenecks observed during the development of trypanosomes in tsetse flies [31]. These bottlenecks may lead to a considerable number of minor genotypes, which are able to escape detection in tsetse mid guts. Furthermore, some minor mid gut trypanosome populations can be amplified in the salivary glands of individual tsetse flies [31]. These minor genotypes could be disseminated to vertebrate hosts, resulting probably in the rapid spread of new genotypes in vertebrate hosts [15]. Another possible outcome of the bottleneck is that some rare variants can be amplified in individual flies and disseminated by them to their vertebrate hosts [32]. If such variants have some selective advantages in mammals, such as altered host range or increased resistance to drugs, this might cause them to become the major variants or genotypes circulating locally [33,34]. Amongst the animals, pigs appear as the species infected by several strains of trypanosomes because 35 different alleles were identified in pigs and about 18 genotypes belonging to the four clusters were also identified in these animals. In addition, almost all genotypes or closely related genotypes were identified in pigs. Pigs therefore appear as the animal species that are able to play an important role in the propagation and the spread of different *T. brucei* genotypes. In this context, the characterization of strains circulating in pigs could give a real indication of the genetic diversity of *T. brucei* circulating in this region. The high number of genotypes circulating in pigs could be linked to the feeding preference of tsetse flies since about 55% of tsetse blood meals collected in this region were from pigs [35].

Out of 71 alleles (multiple and single infections) revealed in this study, only 12 of them were identified in *T. brucei* found in tsetse flies and domestic animals as well. These results indicate a very low number of identical genotypes that circulate between tsetse and domestic animals in this region. This is strengthened by the three genotypes originating from animals that cluster with those found in tsetse (Figure 1). The presence, in each host (tsetse and animals), of several trypanosome genotypes suggests the heterogeneous nature of *T. brucei* strains circulating in the Fontem HAT focus.

The dendrogram of Figure 1 shows a clustering of *T. brucei* genotypes into four clusters. The genotypes belonging to the same cluster can be considered as closely related strains or closely related genotypes. The identification of several genotypes within the same cluster illustrates the genetic differentiation between closely related strains as reported previously by Simo *et al*. [9], for *T. brucei gambiense*. Each cluster can be linked to specific hosts of trypanosomes. For instance, cluster 1 contained strains found mainly in tsetse flies. Within this cluster about 10 different genotypes were identified for 44 samples; thus confirming the low genetic diversity between *T. brucei* circulating in tsetse flies. These results could be explained in part by the low life expectancy of tsetse flies (cannot live for more than four months). In such a context, tsetse flies cannot accumulate infections in the mid-guts like mammals, which are able to do it in the blood or other corporal fluids. The presence of three samples from pigs and goat in the cluster 1 confirms the circulation of closely related genotypes between tsetse flies and mammals. In other clusters where the genetic diversity is high with for instance about 12 genotypes identified in cluster 2 for 18 samples, all *T. brucei* strains came from mammals. This higher genetic diversity observed for the strains of *T. brucei* that circulate in animals could be explained by the longevity of animals in the tsetse infested region. Indeed, most animals sampled in this region have spent at least three months in the region. During this period, these animals had the possibility to be infected by different strains of *T. brucei* due to cumulative and sequential transmission of different genotypes of *T. brucei*.

In order to determine if *T. brucei* populations can be sub-structured on the basis of the origin of samples, the *F*_ST_ was evaluated by considering a subpopulation as all samples originating from the same host or from the same village. Our results show high value of *F*_ST_ between tsetse flies and all animal species. This suggests that most of *T. brucei* strains circulating in tsetse flies are genetically different from strains circulating in domestic animals. Between trypanosomes coming from sheep and goat, the *F*_ST_ value was low (0.0317) and statistically not significant, thus suggesting closely related genotypes between these animals. This hypothesis is strengthened by the presence of genotypes from sheep and goat in the same clusters, mainly in cluster 3 and 4 (Figure 1). The *F*_ST_ values between pigs and other species is moderate and statistically significant. This suggests also that some strains circulating in pigs were genetically different from those found in other hosts. Our results are in line with previous observations of MacLeod *et al*. [30] who reported that host selection is an important determinant of the population structure of *T. brucei*, as particular genotypes of trypanosome are better adapted to survival within different mammalian hosts. It appears that within *T. brucei* subpopulations, each host must be considered as a separate entity. Despite the fact that the sample size was low for some subpopulations like for instance goat, with only eight samples, the results obtained here suggest a sub-structuration of *T. brucei* according to trypanosomes’ hosts.

Looking at the *F*_ST_ between villages (Table 6), some values were low and statistically not significant, suggesting little genetic differentiation or no sub-structuration between *T. brucei* circulating in tsetse flies and animals of some villages of the Fontem focus. However, high and significant *F*_ST_ values between villages like Agong and Folepi (*F*_ST_ = 0.314; P = 0.0001), Folepi and Besali (FST = 0.308; P = 0.0002) indicates that, even in the same focus, considerable genetic differentiation can be observed between *T. brucei* strains of different villages. Such genetic differentiation may have a real impact on the transmission and the spread of the disease like for instance the most pathogenic strains.

## Conclusion

Microsatellite markers enabled us to better understand the distribution of *T. brucei* genotypes in tsetse flies and different species of domestic animals of the Fontem HAT focus of southern Cameroon. Amongst different hosts, pig appeared as the animal species, which is infected by several genotypes of *T. brucei*. The data generated in this study enabled us to cluster *T. brucei* genotypes according to hosts. They also showed a sub structuration of *T. brucei* according to infected animals and sometimes villages. Our results have considerable importance for the understanding of the transmission and the spread of *T. brucei* genotypes.

## Competing interests

The authors declare that they have no competing interests.

## Authors’ contributions

GS participated in the conception of the study, design of experiments, collected field data and draft the manuscript. NGR collected field data, performed molecular analyses and drafted the manuscript. TTMT helped in the population genetics analysis and the draft the manuscript. GC participated in the conception of the study and helped to draft the manuscript. FN participated in the conception of the study, collected field data and helped to draft the manuscript. TA participated in the conception of the study and helped to draft the manuscript. All authors read and approved the final manuscript.

## Supplementary Material

Additional file 1: Figure S1 Frequency of alleles for each microsatellite locus and for each host of *T. brucei*. Click here for file
