# Supplementary figures and images for: Population genetics of Trypanosoma brucei circulating in Glossina palpalis palpalis and domestic animals of the Fontem sleeping sickness focus of Cameroon
Source: Parasit Vectors. 2014 Apr 1;7:156. doi: 10.1186/1756-3305-7-156 (PMC4022266; doi:10.1186/1756-3305-7-156)

Figure S1: Frequency of alleles for each microsatellite locus and for each host of *T. brucei*


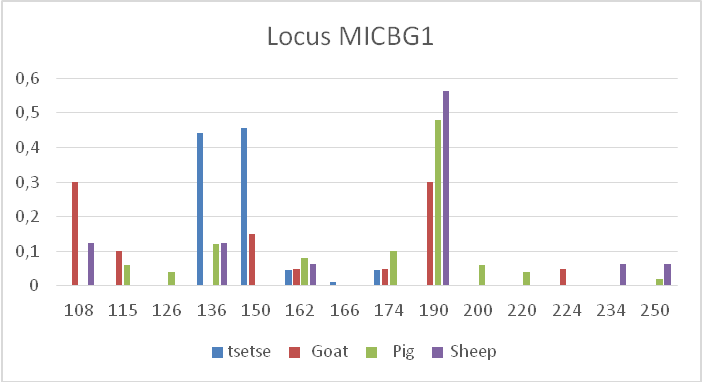


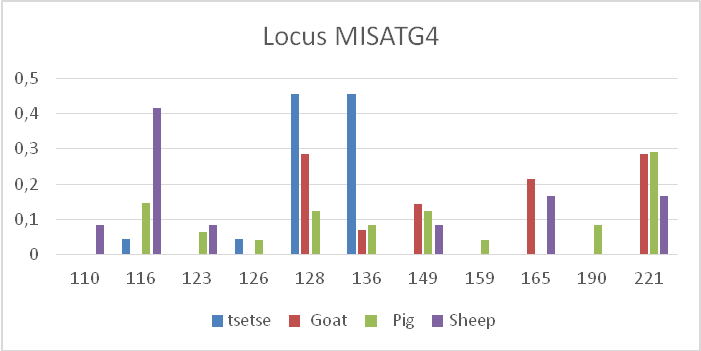


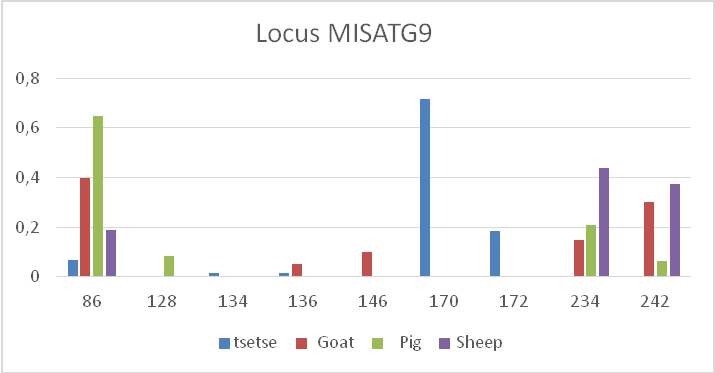


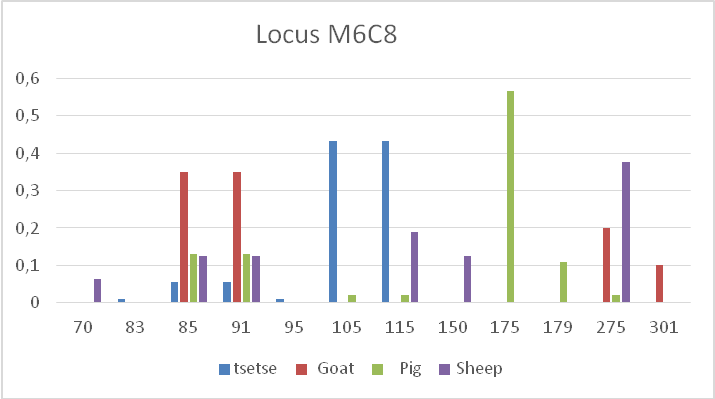


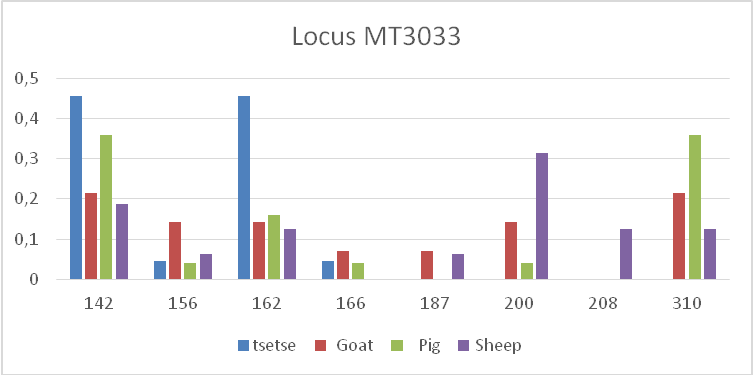

Supplement: Additional file 1: Figure S1 — Frequency of alleles for each microsatellite locus and for each host of T. brucei. [file 1756-3305-7-156-S1.doc]
